# Data report about course on underlying cause-of-death coding (ICD-10): the case virtual learning environment of the Brazilian health system

**DOI:** 10.3389/fdgth.2026.1648954

**Published:** 2026-02-04

**Authors:** Aldiney J. Doreto, António M. Teixeira, Janaina L. R. S. Valentim, João P. Q. Santos, Talita K. de B. Pinto, Yluska M. M. B. Mendes, Aline P. Dias, Karla M. D. Coutinho, Ednara N. Gonçalves, Andréa S. Pinheiro, Felipe Fernandes, Natalia A. N. Batista, Karilany D. Coutinho, Ricardo A. M. Valentim

**Affiliations:** 1 Laboratory for Technological Innovation in Health, Federal University of Rio Grande do Norte, Natal, Brazil; 2 University of Minho (UMinho)/Open University of Portugal (UAb), Lisbon, Portugal; 3 Laboratory of Distance Education and Learning, Open University of Portugal (UAb), Lisbon, Portugal; 4 Advanced Nucleus for Technological Innovation (NAVI), Federal Institute of Rio Grande do Norte (IFRN), Natal, Brazil; 5 Secretariat of Health Surveillance and Environment of the Brazilian Ministry of Health, Brasilia, Brazil; 6 Health Sciences Graduate Program, Federal University of Rio Grande do Norte, Natal, Brazil; 7 Health Sciences Research Unit: Nursing (UICISA: E), Nursing School of Coimbra (ESEnfC), Coimbra, Portugal; 8 Social Sustainability and Development Graduate Program, Open University of Portugal, Lisbon, Portugal; 9 Department of Biomedical Engineering, Federal University of Rio Grande do Norte, Natal, Brazil

**Keywords:** massive open online courses (MOOC), lifelong learning in health, massive learning, cause-of-death, health systems

## 1 Introduction

The process of reporting and coding the cause of death is a vital part of health systems [1, 2]. It provides essential information for monitoring and understanding the main causes of death in a population, which is why it is primordial in the context of epidemiological surveillance. In Brazil, this process is conducted through the Mortality Information System (SIM) of the Brazilian Ministry of Health (MoH). The SIM was established in the 1970s to maintain and manage the country’s death records [3, 4]. Within Brazil’s National Health Surveillance Policy [5], the SIM platform aims to deliver mortality data across all sectors of the health system and to continuously develop health indicators for the entire country [6–8].

The Death Certificate (DC) is the foundational document for the SIM platform and is mandatory for certifying deaths in Brazil [9, 10]. The DC contains crucial information necessary for formulating and planning public health actions based on the specific needs of the population. To accomplish these objectives, physicians must be dedicated and committed to maintaining the accuracy, integrity, and reliability of the information provided in the DC. In Brazil, physicians are solely responsible for completing and ensuring the accuracy of the information contained in the death certificate.

An internationally standardized list of categorized codes for diseases and medical conditions is employed to generate the information in the medical certificate of the DC. In addition, instructions on medical certification and guidelines for classifying and determining the cause of death are utilized [11, 12]. Based on this information and the guidelines, the underlying cause of death is coded and entered into the Mortality Information System. The codes are based on international guidelines utilizing the International Classification of Diseases (ICD) [13–15]. Presently, Brazil uses the tenth version of the classification (ICD-10) to code and select the underlying cause of death, defined as the “disease or injury that initiated a succession of events and ended in death or, in the case of accidents or violence, the circumstances thereof” [16].

In Brazil, the coding of the underlying cause of death is manually performed by coders. These are professionals with diverse backgrounds working in State or Municipal health secretariats. Referred to as “coders of the underlying cause of death,” they assign an ICD-10 code to each medical description and apply international selection and/or modification rules to classify the underlying cause of death. Coders play a critical role in ensuring the generation of accurate mortality data nationwide. Such data is crucial for planning, monitoring, and evaluating policies directed at safeguarding and improving public health [17–19].

This activity requires logical reasoning, knowledge, and skills specific to the use of the three volumes of the ICD-10. Moreover, it involves studying the causes for assigning a specific code to each diagnosis described by the physician on the certificate. Selection and/or modification rules are then applied to derive the underlying cause of death [20]. To this end, the professionals are equipped with the ICD-10 volumes, specific local protocols, and the Underlying Cause Selector (SCB), a module included in the Mortality Information System [21]. As of 2024, approximately 4,000 coders were operating in Brazil, registered with the Ministry of Health [22].

However, errors and inaccuracies have been frequently observed in the completion of death certificates in Brazil. These issues compromise the classification of deaths, resulting in the loss of critical data for the health system and hindering the development of effective policy actions in this sector [20, 23, 24]. Moreover, the shortage of qualified professionals to accurately perform coding tasks has also been observed. This may result in errors in selecting the correct codes, resulting in inaccurate cause-of-death information. Additional issues, such as high coder turnover, limited training provision, insufficient supervision, fragmentation, and duplication of information also hinder the generation of mortality data [25].

Notably, the process of reporting the cause of death on the SIM platform is generally not integrated with other health information systems, which hampers data exchange among different institutions [26, 27]. This compromises the quality and accuracy of mortality statistics in Brazil, further exacerbated by data incompleteness. Consequently, it becomes more difficult to effectively analyze and monitor mortality trends [27–29].

To address these shortcomings, the Brazilian MoH adopted a strategy to enhance professional capacity by promoting training courses on the underlying cause of death coding. Originally, the courses lasted 80 h of in-person instruction. Subsequently, the courses were delivered directly at the State Health Secretariats, with the workload reduced to 64 h and then to 40 h due to challenges in excusing professionals from their work duties to participate. Since 2016, the course has been offered in partnership with IFRN/UFRN in a blended format, with a 64 h workload. Due to the COVID-19 pandemic, the course was adapted to a fully online format while maintaining the same workload. In 2023, the course was redesigned into a self-instructional format, encouraging shared responsibility among the States for continuous professional training [22].

In this context, it is important to emphasize the pivotal role of data generated during the coding process in shaping and implementing public health policies, thereby ensuring the accuracy of population mortality statistics [30]. According to Valentim et al. [31], massive health education through technological mediation constitutes an impactful approach in various scenarios, capable of influencing and contributing to the promotion of public health policies. Especially in countries like Brazil—with a continental size of 8,515,767.049 ${\mathrm{km}}^{2}$, a population exceeding 220 million, and over 570,000 physicians distributed nationwide—knowledge dissemination strategies are key to achieving public health policy goals more quickly and effectively [32–34].

Similarly, Moreira Teixeira et al. [18] assert that massive open education through Massive Open Online Courses (MOOCs) can be an effective and sustainable solution for the continuous training of health professionals. Studies on digital learning effectiveness, such as the meta-analysis by Means et al. [35], often indicate that online learning can be as or more effective than traditional methods, provided there is adequate attention to instructional design and human-technology interaction (HTI) [36]. Furthermore, considering the typical challenges and evaluation models of MOOCs, which frequently address issues like low completion rates [37], it is crucial to recognize their potential when specifically applied to continuous professional development (CPD). As such, MOOCs are tools that can contribute to the training required to improve the quality of cause-of-death data input into the Mortality Information System. This, in turn, enhances the understanding of epidemiological trends and the impact of health policies in Brazil.

These deficiencies in the process of cause-of-death reporting and coding in Brazil negatively impact data quality and hamper the ability to monitor and understand the leading causes of death at a national level—an obstacle to the formulation, evaluation, and monitoring of public health policies. For instance, one-third of deaths in Brazil are reported with causes that are not useful for public health analysis, commonly referred to as “garbage codes” (GC) [38].

This scenario reinforces the case for investing in adequate training for health professionals through health education—based interventions, aimed at improving data entry and the completion of death certificates issued by the Brazilian State. Recognizing the difficulties and challenges involved, the Brazilian MoH offered the “Training Course on Underlying Cause-of-Death Coding – ICD-10” to 1,533 professionals nationwide. The aim was to enhance the quality of information recorded in the Mortality Information System.

Given this phenomenon, particularly in the context of the course offered by the Brazilian MoH, this data report aimed to demographically characterize a group of course participants and their perceptions regarding the “Training Course on Underlying Cause-of-Death Coding – ICD-10.” Since this study resulted in a data report, its primary contribution lies in characterizing and sharing the data with health professionals and scholars interested in this topic of global interest, not exclusively within Brazil.

## 2 Materials and methods

### 2.1 Study design and participants

This is a descriptive study of the online learning module of the “Training Course on Underlying Cause-of-Death Coding – ICD-10,” offered at no charge through the Virtual Learning Environment of the Brazilian National Health System (AVASUS). AVASUS is a crucial digital health resource for Brazil’s National Health System (SUS) designed to promote, support, and strengthen continuing health education. As an online learning platform, AVASUS facilitates knowledge transfer, health workforce training, and the implementation of rapid-response strategies to public health emergencies and crises, such as the COVID-19 pandemic [39].

According to open data on the digital platform itself, AVASUS has 426 active courses and has surpassed 1.2 million course participants, totaling more than 3.3 million enrollments. In particular, the online learning module of the “Training Course on Underlying Cause-of-Death Coding – ICD-10” has 1,533 enrollees. These participants were included in the study, and a duly anonymized data set was used for the descriptive analysis. It is worth noting that this course is restricted to specific profiles, meaning it is not open to the general public. Enrollment was subject to the criteria established by Brazil’s MoH. Priority for the available placements was accorded to public sector officials operating within local authorities or administrative regions with the greatest incidence and systematic coding of mortality over the course of the year.

### 2.2 Data acquisition

Data for the descriptive analysis were primarily obtained from four sources: (i) AVASUS [40]; (ii) the National Register of Health Facilities (CNES) [41]; (iii) the Brazilian Occupational Classification (CBO) [42]; and (iv) the Brazilian Institute of Geography and Statistics (IBGE) [43]. All data has been duly anonymized, integrated, and made available through a public repository at https://doi.org/10.5281/zenodo.13984652. This data does not constitute or characterize an experimental study involving human beings, thus excluding the need for approval by a research ethics committee.

AVASUS served as the primary data source for this study, accounting for the majority of the data used in descriptive analysis. Through AVASUS, data was collected from 1,533 instances, i.e., objects representing participants enrolled in the “Training Course on Underlying Cause-of-Death Coding – ICD-10.” For each instance, a set of 39 features or attributes was collected. The main features analyzed in this study include gender, CNES, certificate eligibility, Brazilian region, CBO, and quantitative and qualitative evaluations of the course. It is worth noting that the course was offered on AVASUS and received registrations according on demand from the Brazilian Federative Units (UF), as authorized by Brazil’s MoH. Therefore, the dataset covers the period from the beginning of the course on July 24, 2018, to the data collection on February 22, 2024. It should also be emphasized that the certificate of completion is only granted to participants who fully complete the training, fulfilling 100% of the proposed activities and obtaining a minimum average of 7.0 on a scale of 0 to 10.

Data from the 2022 demographic census released by the IBGE, concerning the Brazilian population and its regions, was collected to support the analysis [43]. The aggregation of the instances with their respective attributes constitutes this study’s dataset, thereby enabling the demographic characterization and profiling of course participants. It also provides essential tools for evaluation, taking into account the course participants’ perspectives and the process of continuing health education mediated by technology through AVASUS.

### 2.3 Data processing

Following data collection, a data engineering process was carried out. Hence, a pipeline, or workflow, was defined to verify, sanitize, transform, and validate the data, thereby generating a structured, secure, and viable dataset for descriptive analysis and the scientific community. The following steps were considered in the data processing pipeline: (i) data quality assessment; (ii) data integration and standardization; (iii) feature extraction; and (iv) feature selection. All steps were performed in an environment configured with the Python 3.10.12 programming language and auxiliary libraries such as numpy, pandas, matplotlib, seaborn, and enelvo.

In step (i), data quality assessment, the dataset was inspected for identification of instances with missing, inconsistent, or noisy values, i.e., values outside normal ranges. No strategies or synthetic data were used to fill in the missing values. Fields with missing values mean that the respective participant, or instance of the dataset, did not complete the course and/or did not evaluate it. Furthermore, no anomalies were found in the data. In step (ii), data integration and standardization, a procedure was conducted to retrieve the CBO code of participants with professional affiliations through the CNES. Then, the CBO codes were associated with their respective instances and integrated into the main dataset as a new attribute. Course participants without formal professional affiliation or who did not provide a CBO code were labeled as “individuals with no formal affiliation.” Specifically for the attribute related to course participants’ gender, a standardization of nomenclatures was necessary, and the following terms were applied: Female, Male, and Not reported.

In step (iii), feature extraction, the attributes related to the region and the descriptive classification of the course participants’ occupations were created. Using the CBO code, as well as the official CBO data source in Brazil [42], a procedure was performed to decode the CBO code and integrate the names of the corresponding occupations into the dataset. In this particular case, a treatment using regular expressions was performed to reduce dispersion between synonymous occupations. For example, the different descriptions of occupations derived from the medical field (i.e., specialty physicians) were treated and aggregated into a single group labeled “physician.”

In the final procedure of step (iii), the “Region” attribute was created from the attribute relating to the course participant’s Federative Unit or State, a value contained in the AVASUS dataset. This attribute was created to group course participants into one of Brazil’s five regions: North, Northeast, Central-West, Southeast, and South, following the country’s political-administrative and regional divisions [44].

In the last step, (iv) feature selection, the attributes of great relevance for the descriptive analysis in this study were defined. At this point, a meticulous review was undertaken to ensure data consistency, coherence, and anonymization, as well as to make the dataset publicly available. All attributes that could identify or track participants were removed from the original dataset. A detailed description of the dataset is available for public consultation in the repository https://doi.org/10.5281/zenodo.13984652.

## 3 Data analysis

The data from the online learning module of the “Training Course on Underlying Cause-of-Death Coding – ICD-10” was analyzed using descriptive statistics, which allows for the fundamental exploration and description of the relevant properties and characteristics of the dataset. The principal resources used were: measures of absolute and relative frequencies; measures of central tendency, mean and median; measures of dispersion or spread, observing the standard deviation (std).

**Figure 1:**
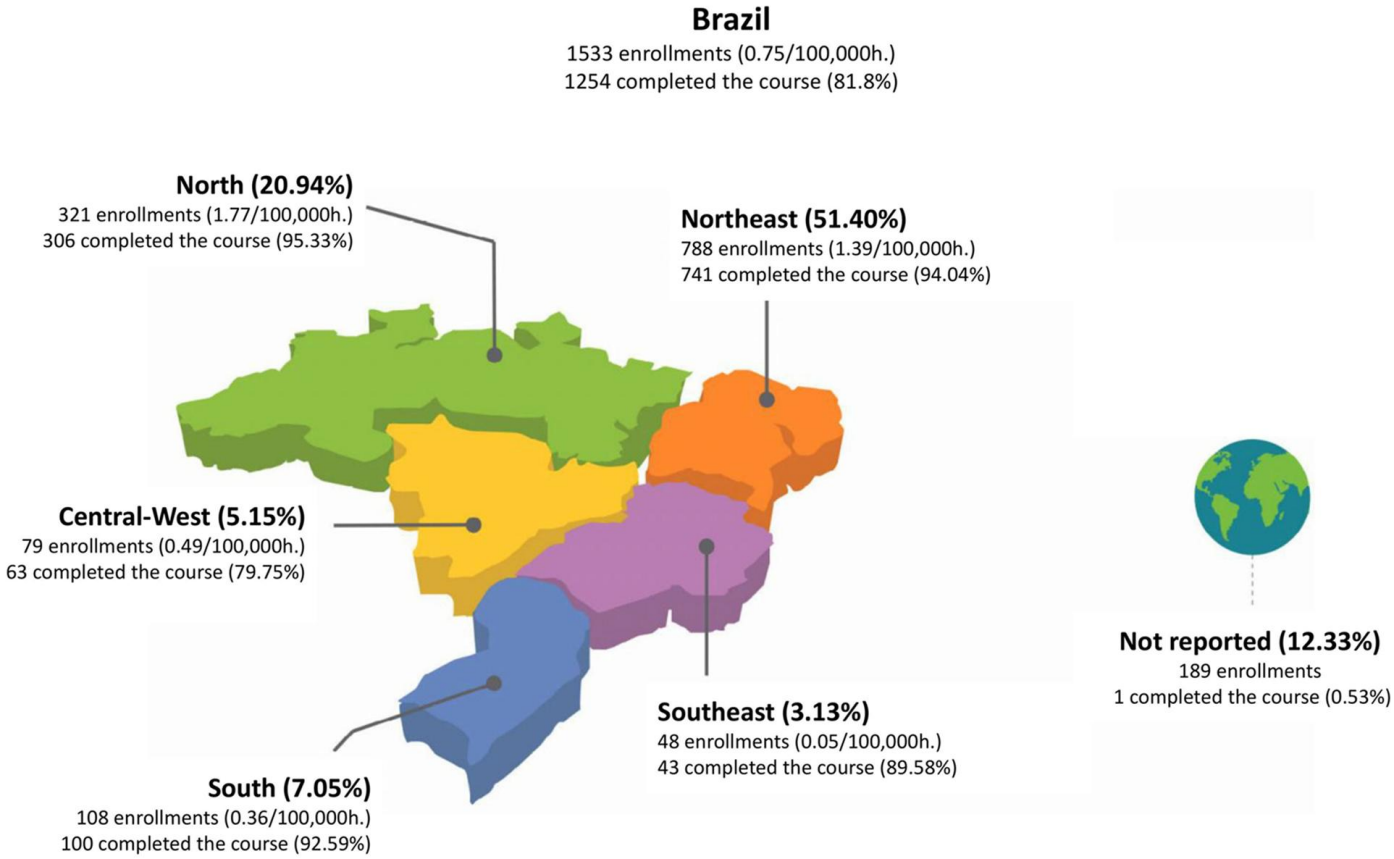
Characterization and geographical distribution of the group of course participants.

Drawing on the analysis model proposed by Valentim et al. [31], Equation 1 was employed to normalize the data related to enrollments and populations in each Brazilian region. Therefore, the coefficient “rate” represents the proportion of each region analyzed (normalized values per 100,000 population). Equation 1 was primarily used to construct Figure 1. The population figures for Brazil’s regions, as well as the country’s population, were elicited from the 2022 demographic census published by the Brazilian Institute of Geography and Statistics (IBGE) [43]. The following notations have been defined for the variables contained in Equation 1:

$$\mathrm{rate}=\left(\frac{x_{\mathrm{target}}}{x_{\mathrm{pop}}}\right)n_{\mathrm{factor}}$$ (1)

were:

rate: variable storing the coefficient for indicators proportional to each region or Brazil;
$x_{\mathrm{target}}$: variable for determining the value associated with the indicators (number of enrollments);
$x_{\mathrm{pop}}$: variable for determining the population of each region;
$n_{\mathrm{factor}}$: variable for determining the proportionality factor.

## 4 Descriptive analysis

The online learning module of the “Training Course on Underlying Cause-of-Death Coding – ICD-10,” available on AVASUS https://avasus.ufrn.br/local/avasplugin/cursos/curso.php?id=231, recorded 1,533 enrollments, of which 1,023 identified as female (66.73%) and 315 as male (20.55%). Moreover, 195 course participants did not report gender (12.72%). Considering the overall group, 1,254 (81.8%) participants were eligible for certification in the online module of the course. As of the day the data was exported, a group of 279 participants (18.2%) had not completed 100% of the online activities.

In Figure 1, it can be seen that the course had enrolments from all five Brazilian regions. The largest number of participants was from the Northeast region, totaling 788 people (51.4%). Despite the prioritisation of placements for local authorities or administrative regions with the highest incidence of mortality coding throughout the year, the significant number of enrolments in the Northeast region can be partly explained by the historical deficit in training opportunities in previous years, as well as by the support provided by local public administration.

**Figure 2:**
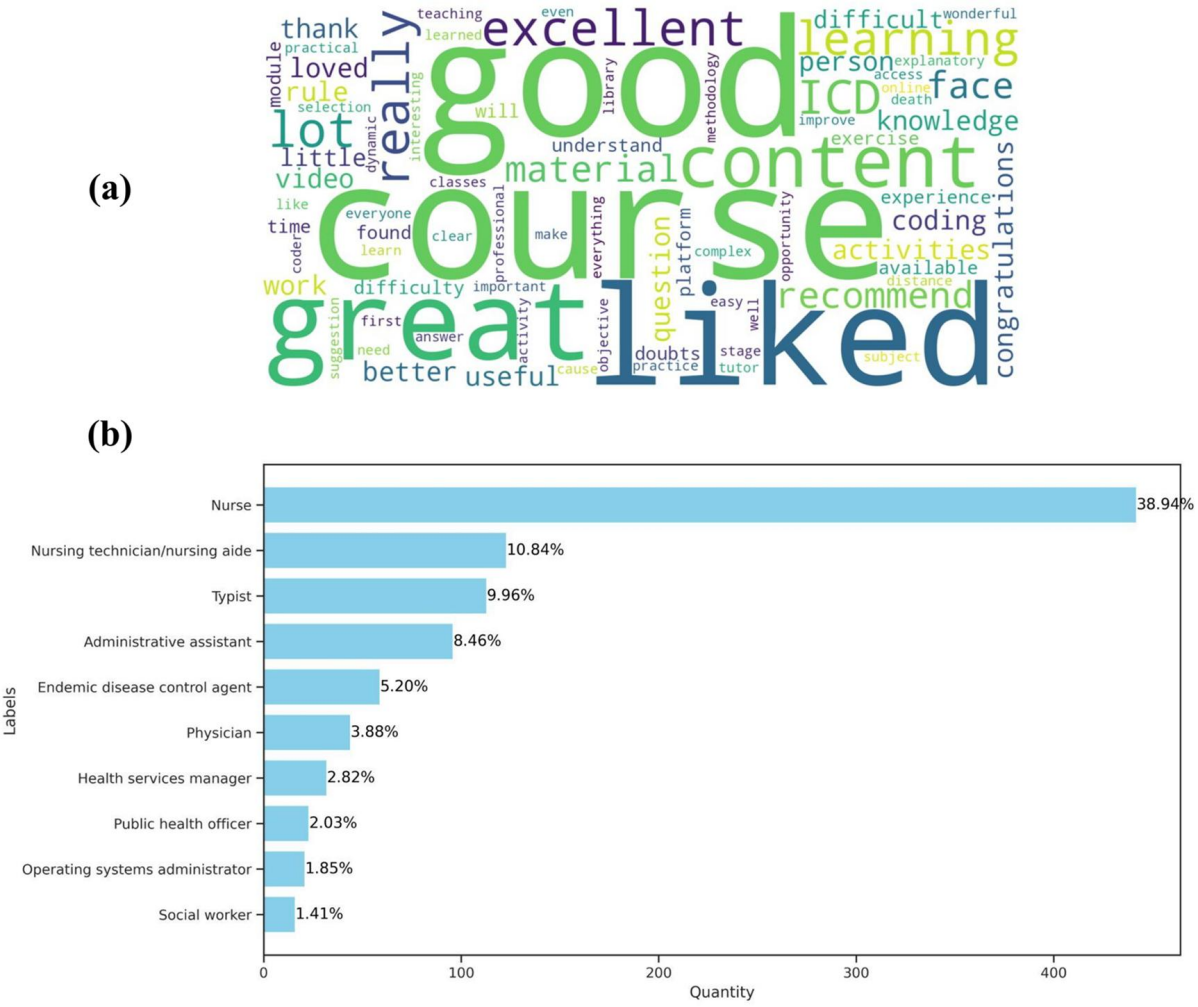
Summary of repository data analysis. **(a)** Wordcloud of comments from course evaluations. **(b)** Distribution of the ten most frequent occupations among the group of course participants working.

The online learning module of the “Training Course on Underlying Cause-of-Death Coding – ICD-10” was evaluated by a group of 927 course participants (60.47%). On a satisfaction scale ranging from 1 to 5, the course obtained an average score of 4.8 (median = 5 and standard deviation = 0.56). The same group also evaluated the course by submitting written comments in a specific field on AVASUS. Using the analysis strategy based on word clouds for visual text representation, Figure 2a was created from the comments written by course participants.

From the perspective of the course participants’ professional profile, a group of 1,135 professionals (74.04%) were found to have different occupations. Figure 2b shows the ten most frequent occupations among course participants working. At the top of the list, it is clear that the occupation “nurse” is the most frequent among course participants, with a total of 442 (38.94%) professionals in this field. Following this, also in the field of nursing, are nursing technicians and nursing aides, totaling 123 (10.84%) participants in this occupation.

## Data Availability

The datasets presented in this study can be found in online repositories. The names of the repository/repositories and accession number(s) can be found below: The datasets analyzed for this study can be found in the Zenodo: https://doi.org/10.5281/zenodo.13984652.
